# NF-KappaB Pathway Is Involved in Bone Marrow Stromal Cell-Produced Pain Relief

**DOI:** 10.3389/fnint.2018.00049

**Published:** 2018-10-16

**Authors:** Wei Guo, Satoshi Imai, Jia-Le Yang, Shiping Zou, Huijuan Li, Huakun Xu, Kamal D. Moudgil, Ronald Dubner, Feng Wei, Ke Ren

**Affiliations:** ^1^Department of Neural and Pain Sciences, School of Dentistry & Program in Neuroscience, University of Maryland, Baltimore, MD, United States; ^2^Department of Clinical Pharmacology & Therapeutics, Kyoto University Hospital, Kyoto, Japan; ^3^Department of Neurology, The 3rd Affiliated Hospital of Sun Yat-sen University, Guangzhou, China; ^4^Department of Advanced Oral Sciences and Therapeutics, University of Maryland School of Dentistry, Baltimore, MD, United States; ^5^Department of Microbiology & Immunology, University of Maryland, Baltimore, MD, United States

**Keywords:** orofacial pain, tendon ligation, mesenchymal stromal cells, BAY 11-7082, chemokine, rostral ventromedial medulla

## Abstract

Bone marrow stromal cells (BMSCs) produce long-lasting attenuation of pain hypersensitivity. This effect involves BMSC’s ability to interact with the immune system and activation of the endogenous opioid receptors in the pain modulatory circuitry. The nuclear factor kappa B (NF-κB) protein complex is a key transcription factor that regulates gene expression involved in immunity. We tested the hypothesis that the NF-κB signaling plays a role in BMSC-induced pain relief. We focused on the rostral ventromedial medulla (RVM), a key structure in the descending pain modulatory pathway, that has been shown to play an important role in BMSC-produced antihyperalgesia. In Sprague-Dawley rats with a ligation injury of the masseter muscle tendon (TL), BMSCs (1.5 M/rat) from donor rats were infused i.v. at 1 week post-TL. P65 exhibited predominant neuronal localization in the RVM with scattered distribution in glial cells. At 1 week, but not 8 weeks after BMSC infusion, western blot and immunostaining showed that p65 of NF-κB was significantly increased in the RVM. Given that chemokine signaling is critical to BMSCs’ pain-relieving effect, we further evaluated a role of chemokine signaling in p65 upregulation. Prior to infusion of BMSCs, we transduced BMSCs with *Ccl4* shRNA, incubated BMSCs with RS 102895, a CCR2b antagonist, or maraviroc, a CCR5 antagonist. The antagonism of chemokines significantly reduced BMSC-induced upregulation of p65, suggesting that upregulation of p65 was related to BMSCs’ pain-relieving effect. We then tested the effect of a selective NF-κB activation inhibitor, BAY 11-7082. The mechanical hyperalgesia of the rat was assessed with the von Frey method. In the pre-treatment experiment, BAY 11-7082 (2.5 and 25 pmol) was injected into the RVM at 2 h prior to BMSC infusion. Pretreatment with BAY 11-7082 attenuated BMSCs’ antihyperalgesia, but post-treatment at 5 weeks post-BMSC was not effective. On the contrary, in TL rats receiving BAY 11-7082 without BMSCs, TL-induced hyperalgesia was attenuated, consistent with dual roles of NF-κB in pain hypersensitivity and BMSC-produced pain relief. These results indicate that the NF-κB signaling pathway in the descending circuitry is involved in initiation of BMSC-produced behavioral antihyperalgesia.

## Introduction

Chronic orofacial pain affects approximately 20 percent of the population and is a major health problem (Isong et al., 2008; Hargreaves, 2011). The most common persistent orofacial pain condition, temporomandibular joint disorders, affects the musculoskeletal and joint tissues, is heterogeneous in origin, and often not successfully treated. Bone marrow stromal cells (BMSCs) are a major type of mesenchymal stem (stromal) cells that can differentiate into lineages of the mesenchyme such as osteoblasts, chondrocytes and adipocytes *in vitro* (Pittenger et al., 1999). One important property of BMSCs is their potential for immune regulation (Davies et al., 2017), which have attracted great interest in exploring their therapeutic use in a variety of disease conditions including chronic pain. Both preclinical and clinical studies have shown pain-relieving or antihyperalgesic effect of BMSCs (Black et al., 2007; Abrams et al., 2009; Guo et al., 2011, 2017; Siniscalco et al., 2011; Roh et al., 2013; Sacerdote et al., 2013; Franchi et al., 2014; Vickers et al., 2014; Chen et al., 2015; Pettine et al., 2015, 2016; Watanabe et al., 2015; Evangelista et al., 2018). In a rat model of myogenic orofacial pain involving the ligation injury of one tendon of the masseter muscle (Guo et al., 2010), we have shown that systemic infusion of BMSCs produced long-term attenuation of persistent pain in both male and female rats, indicated by inhibition of thermal and mechanical nociception and pain aversion (Guo et al., 2011, 2016). We further showed that BMSC-produced antihyperalgesia required their interactions with host immune cells and activation of mu opioid receptors (MOR) in the pain-modulatory circuitry (Guo et al., 2014, 2017). Our findings call attention on immune regulation as a mechanism of BMSCs’ therapeutic effects.

The nuclear factor kappa B (NF-κB) protein complex is a transcription factor that is found in almost all cell types and controls the transcription of multiple genes involved in immunity (Bonizzi and Karin, 2004). NF-κB is a dynamic nuclear transcription factor that can be activated by a variety of stimuli. Mesenchymal stromal cells promote neuroprotection via NF-κB-mediated gene transcription (Walker et al., 2010). Interestingly, tumor necrosis factor (TNF) produces neuroprotection and stimulates MOR expression in neurons involving the NF-κB pathway (Tamatani et al., 1999; Kraus et al., 2003; Fang et al., 2018). The activation of NF-κB correlates with increased anti-inflammatory cytokine interleukin (IL)-10 in human whole blood cell culture (Al-Hanbali et al., 2009) and induces IL-10 expression in human monocytes (Pilette et al., 2010). We reason that the NF-κB-involved signaling may play a role in BMSC-induced pain relief. This hypothesis was tested in the present study. We focused on the central mechanisms involving rostral ventromedial medulla (RVM), a key structure in the descending pain modulatory pathway, that has been shown to play an important role in BMSC-produced antihyperalgesia (Guo et al., 2011, 2017). We showed that there was an upregulation of p65 of NF-κB in the RVM after BMSC treatment, and injection of the NF-κB activation inhibitor into the RVM attenuated BMSC-produced antihyperalgesia.

## Materials and Methods

### Animals

Male Sprague-Dawley rats, ≈8-week old at the time of surgery, were used (Envigo-Harlan). Animals were housed on the 9th floor of the University of Maryland School of Dentistry. The facility is an approved, registered research site (USDA #MD-R-118) and accredited by AAALAC. Animals were kept under controlled environment conditions (≈22°C), relative humidity 40%–60%, 12 h/12 h light-dark cycles, and food and water *ad libitum*. The animals’ conditions were monitored continuously throughout the course of studies, which include body weight, grooming, locomotion, ambulant activity and condition of the wound. The behavioral studies involve stimulation that produces only momentary additional pain/or discomfort and the rats can escape from the stimuli at any time.

All surgical procedures were performed under pentobarbital sodium (20–50 mg/kg i.p.) anesthesia. Ligation of the tendon (TL) of the anterior superficial part of the rat masseter muscle was achieved via an intraoral approach as described elsewhere (Guo et al., 2010). Briefly, on the left intraoral site, a 5-mm long incision was made posterior-anteriorly lateral to the gingivobuccal margin in the buccal mucosa, beginning immediately next to the first molar. The tendon of the anterior superficial part of the rat masseter muscle was gently freed and tied with two chromic gut (4.0) ligatures, 2-mm apart. Animals were randomly assigned to experimental groups. The number of animals per group was determined by our previous studies and a power analysis. All experiments were carried out in accordance with the National Institutes of Health (NIH) Guide for the Care and Use of Laboratory Animals (NIH Publications No. 80-23) and approved by the Institutional Animal Care and Use Committee, University of Maryland School of Dentistry/Medicine.

### BMSC Procedures

BMSCs were obtained from donor rats as described (Shen et al., 2006; Guo et al., 2011). The rats were euthanized with CO_2_ and both ends of the tibiae, femurs and humerus were cut off by scissors. A syringe fitted with an 18-gauge needle was inserted into the shaft of the bone and bone marrow was flushed out with culture medium (alpha-modified Eagle medium, Gibco, Carlsbad, CA, USA; 10% fetal bovine serum, Hyclone, Logan, UT, USA). The bone marrow was then mechanically dissociated and the suspension passed through a 100-μm cell strainer to remove debris. The cells were incubated at 37°C in 5% CO_2_ in tissue-culture flasks (100 × 200 mm; Sarstedt, Nümbrecht, Germany), and non-adherent cells removed by replacing the medium. At day 7, when the cultures reached 80% confluence, the cells were washed with PBS and harvested. The cell numbers were calculated by the Hemocytometer. For intravenous administration, 1.5 × 10^6^ cells (1.5 M) in 0.2 ml PBS were slowly injected into one tail vein of the anesthetized rat over a 2-min period using a 22-gauge needle. The property of expanded cells was assessed by flow cytometry with conventional markers (Guo et al., 2011). Flow cytometry analyses were performed at the University of Maryland GreenBaum Cancer Center Shared Flow Cytometry Facility.

### Behavioral Testing

All behavioral tests were conducted under blind conditions. Mechanical sensitivity of the orofacial region was assessed as described (Ren, 1999; Guo et al., 2010). A series of calibrated von Frey filaments were applied to the skin above the injured tendon or the corresponding contralateral side. An active withdrawal of the head from the probing filament was defined as a response. Each von Frey filament was applied five times at intervals of 5–10 s. The response frequencies [(number of responses/number of stimuli) × 100%] to a range of von Frey filament forces were determined and a stimulus-response frequency (S-R) curve plotted. After a non-linear regression analysis, an EF_50_ value, defined as the effective von Frey filament force (g) that produces a 50% response frequency, was derived from the S-R curve (Prism, GraphPad; Guo et al., 2004). A leftward shift of the S-R curve, resulting in a reduction of EF_50_, occurred after TL, which suggests the development of mechanical hypersensitivity, including allodynia and hyperalgesia.

### Western Blot

Rats were anesthetized with isoflurane (3%) and quickly decapitated. The brainstem tissue block that included the RVM was harvested by taking punches with a 15-gauge needle. The tissues were homogenized in solubilization buffer (50 mM Tris.HCl, pH 8.0; 150 mM NaCl, 1 mM EDTA, 1% NP40, 0.5% deoxycholic acid, 0.1% SDS, 1 mM Na_3_VO_4_, 1 U/ml aprotinin, 20 μg/ml leupeptin, 20 μg/ml pepstatin A). The homogenate was centrifuged at 20,200× *g* for 10 min at 4°C. The supernatant was removed. The protein concentration was determined using a detergent-compatible protein assay with a bovine serum albumin standard. For detecting the immunoreactivity with near-infrared fluorescence using the Odyssey Infrared Imaging System (Odyssey^®^CLx, LI-COR, Lincoln, NE, USA), 50-μg protein samples were denatured by boiling for 5 min and loaded onto 4%–20% Bis-Tris gels (Invitrogen). After electrophoresis, proteins were transferred to nitrocellulose membranes. The membranes were blocked for 1 h with Odyssey Blocking Buffer and then incubated with primary antibodies (Anti-NF-κB, p65 subunit (Cell Signaling) diluted in Odyssey Blocking Buffer at 4°C overnight, followed by washing with PBS containing 0.1% Tween 20 (PBST) three times. The membranes were then incubated for 1 h with IRDye800CW-conjugated goat anti-rabbit IgG and IRDye680-conjugated goat anti-mouse IgG secondary antibodies (LI-COR) diluted in Odyssey Blocking Buffer. The blots were further washed three times with PBST and rinsed with PBS. Proteins were visualized by scanning the membrane with 700 and 800-nm channels. The loading and blotting of the amount of protein was verified by reprobing the membrane with anti-β-actin and with Coomassie blue staining.

### Immunohistochemistry

Rats were deeply anesthetized with pentobarbital sodium (100 mg/kg, i.p.) and perfused transcardially with 4% paraformaldehyde in 0.1 M phosphate buffer at pH 7.4. The brain stem was removed, post-fixed, and transferred to 25% sucrose (w/v) for cryoprotection. Transverse sections (20-μm) were cut with a cryostat. The free-floating sections were incubated with relevant antibodies with 1% normal goat sera and 0.3% Triton X-100 overnight at 4°C. After washes in PBS, the sections were incubated with relevant IgGs conjugated to Cy3 or Cy2 (1:500; Jackson ImmunoResearch, West Grove, PA, USA) for 4 h at room temperature or overnight at 4°C. Double immunofluorescent staining was performed for p65 with NeuN (Chemicon), glial fibrillary acidic protein (GFAP; Chemicon) or CD11b (AbD Serotec, Novus). Following washes, the stained sections were mounted on gelatin-coated slides and coverslipped with Vectashield (Vector Laboratories). Slides were examined with a Nikon fluorescence microscope and images were captured with a CCD Spot camera. Control sections were processed with the same method except that the primary antisera are omitted.

### Brainstem Microinjections

Rats were anesthetized with 2%–3% isoflurane in a gas mixture of 30% O_2_ balanced with 70% nitrogen and placed in a Kopf stereotaxic instrument (Kopf Instruments, Tujunga, CA, USA). A midline incision was made after infiltration of lidocaine (2%) into the skin. A midline opening was made in the skull with a dental drill for inserting an injection needle into the target site. The coordinates for the RVM were: 10.5–11.5 mm caudal to the Bregma, midline and 9.0 mm ventral to the surface of the cerebellum (Paxinos and Watson, 2014). Microinjections were performed by delivering drug solutions slowly over a 10-min period using a 500 nl Hamilton syringe with a 32-gauge needle. The injection needle was left in place for at least 15 min before being slowly withdrawn. The wound was closed and animals were returned to their cages after recovering from anesthesia. For histology verification of the injection site, 30-μM coronal brainstem sections were stained with Neurotrace™ 500/525 Green fluorescent Nissl Stain (Invitrogen; 1:500 for 20 min).

### RNAi

*Ccl4* shRNA (Accession Number NM_053858.1 CDS, target sequence: 90: TCCCACTTCCTGCTGCTTCTCTTACACCT) was transduced into cultured BMSCs [*Ccl4* RNAi lentivirus (piLenti-siRNA-GFP, abm^®^ Richmond, BC, Canada)]. BMSCs were plated onto 10-cm plate before transduction. When they reached 80% confluence 5 ml medium (without serum) was added with 4 μl polybrene (8 μg/ml) and 50 μl of ViralPlus Transduction Enhancer G698 (abm). Eighty-microliter *Ccl4* shRNA lentivirus or control shRNA lentivirus was then added to the plate. Cells were incubated at 37°C with 5% CO_2_ and collected at 72 h following transduction. Successful transduction and knockdown of *Ccl4* were verified (Guo et al., 2017).

### Drugs

All drugs were purchased: NF-κB activation inhibitor **BAY 11-7082** [(E)-3-(4-Methylphenylsulfonyl)-2-propenenitrile] MW 207.25, CAS Number 19542-67-7 (Calbiochem), CCR2 receptor antagonist **RS-102895** hydrochloride {1′-[2-[4-(Trifluoromethyl)phenyl]ethyl]-spiro[4H-3,1-benzoxazine-4,4′-peperidin]-2(1H)-one} MW 426.86, CAS number 300815-41-2 (Sigma-Aldrich), and CCR5 antagonist **maraviroc** {4,4-Difluoro-N-[(1S)-3-[(3-exo)-3-[3-methyl-5-(1-methylethyl)-4H-1,2,4-triazol-4-yl]-8-azabicyclo[3.2.1]oct-8-yl]-1-phenylpropyl]cyclohexanecarboxamide} MW 513.68, CAS Number 376348-65-1 (R&D). Drugs were dissolved in 5% dimethyl sulfoxide and saline.

### Data Analysis

Data are presented as means ± SEM. Analysis of variance (ANOVA) and the *post hoc* Tukey test was performed for protein data. One- or two-way ANOVA with repeated measures was used for comparison of EF_50_s, followed by *post hoc* test with corrections for multiple comparisons. *P* < 0.05 was considered significant for all cases.

## Results

**Figure 1:**
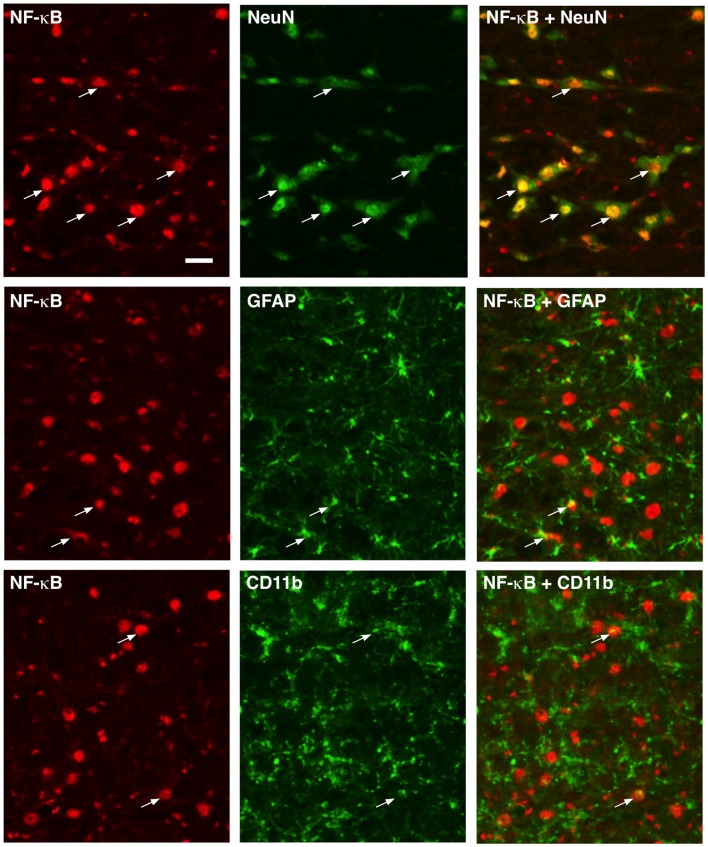
Localization of p65 of nuclear factor kappa B (NF-κB) in rostral ventromedial medulla (RVM). Numerous neurons (top row) exhibited p65 immunoreactivity shown by double immunofluorescence with NeuN. Few double-labeling profiles of p65 were shown with glial fibrillary acidic protein (GFAP; middle row; astrocytes) and CD11b (bottom row; microglia). Examples of double labeled profiles are indicated by arrows. Scale = 40 μm.

NF-κB functions as dimers formed from five-member proteins (p50, p52, p65, RelB and c-Rel; Bonizzi and Karin, 2004). The p50/p65 dimer is the most abundant and considered canonical. P50 contains a nuclear localization sequence, provides DNA binding, and interacts with the IκB (NF-κB inhibitory proteins), while p65 has a transcription activation domain and is a transcriptional activator. Immunostaining of p65 showed wide-spread distribution in RVM tissues (Figure 1). Double immunofluorescence labeling indicated p65 expression in neurons, as shown by extensive co-localization with NeuN, a neuronal marker (Figure 1, Top row). In contrast, fewer scattered double-labeling profiles were seen with GFAP, an astrocyte marker (Figure 1, Middle row), and CD11b, a microglia marker (Figure 1, Bottom row).

**Figure 2:**
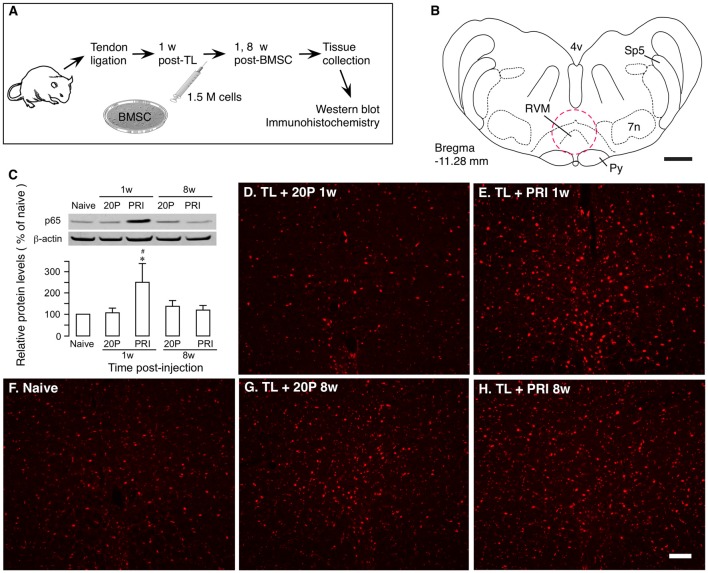
Upregulation of NF-κB by bone marrow stromal cells (BMSCs). **(A)** Flowchart of the experiment. **(B)** A drawing of brainstem transverse section illustrating RVM (Paxinos and Watson, 2014). Dashed circle indicates the area punched for analysis. 4v, fourth ventricle; 7n, facial nucleus; Py, pyramidal tract; RVM, rostral ventromedial medulla; Sp5, spinal trigeminal tract. Scale = 1 mm. **(C)** Effect of BMSCs on p65 of nuclear factor kappa B (NF-κB) in RVM. RVM tissues were collected at 1 week and 8 weeks after the BMSC injection. Total proteins isolated and separated. An example of the blot is shown on top and the relative protein levels are shown in the bottom histogram. β-actin was a loading control. p65 of NF-κB was upregulated at 1 week after injection of primary (PRI) BMSC. *p<0.05 vs. Naïve; ^#^*p* < 0.05 vs. 20P (20-passage) BMSCs. **(D–H)** Immunostaining of p65 in RVM. Note an apparent increase in immunoreactivity in TL + PRI 1 week. **(E)** Scale = 0.1 mm.

Ligation injury of the masseter muscle tendon was produced to assess BMSC-produced effects as described previously (Guo et al., 2010, 2011). Masseter TL induces nociceptive hypersensitivity lasting for months. At 1 week after TL, 1.5 × 10^6^ BMSCs were infused into one tail vein and at 1 and 8 weeks after BMSC infusion, RVM tissues were collected for western blot and immunohistochemistry (Figures 2A,B). We have shown that primary, but not 20-passage (20P) BMSCs, attenuate persistent pain hypersensitivity (Guo et al., 2011). Thus, 20P BMSCs were used as a control. Western blot showed that p65 of NF-κB was significantly upregulated at 1 week after injection of primary BMSCs, compared to naïve and 20P BMSC-treated TL rats (Figure 2C). There was no change in p65 at 8 weeks after BMSC infusion. Similar changes in p65 immunostaining were observed in RVM (Figures 2D–H).

**Figure 3:**
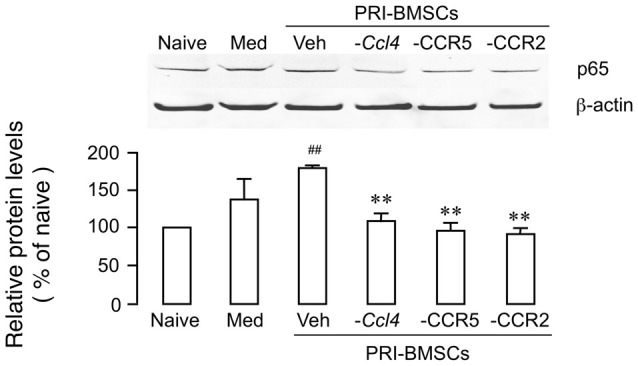
Attenuation of BMSC-induced upregulation of p65 by antagonism of chemokine signaling associated with BMSCs’ pain-relieving effect. Prior to infusion of BMSCs, *Ccl4* was knocked down from primary BMSCs by transduction with *Ccl4* shRNA, CCR2 was blocked by pretreatment of BMSCs with RS 102895 (10 μM for 24 h), a CCR2b antagonist, and CCR5 was blocked by pretreatment of BMSCs with maraviroc (200 nM for 24 h), a CCR5 antagonist. Culture medium (Med) was a control for BMSCs. ^##^*p* < 0.01 vs. Naïve; **p<0.01 vs. Veh. *N* = 4.

We have shown previously that chemokine signaling is critical to BMSCs’ pain-relieving effect (Guo et al., 2017). Compared to primary BMSCs, a number of chemokines/receptors were significantly down-regulated in 20P BMSCs including CCL4, CCR2 and CCR5, which underlies their inability to produce antihyperalgesia. Knock-down of CCL4 from BMSCs or blockage of CCR2 or CCR5 reverse BMSC-produced upregulation of MOR and antihyperalgesia (Guo et al., 2017, 2018). To evaluate a role of chemokine signaling in p65 upregulation, we performed the same manipulations prior to infusion of BMSCs, including transduction of primary BMSCs with *Ccl4* shRNA, incubation of primary BMSCs with RS 102895 (10 μM for 24 h), a CCR2b antagonist, or maraviroc (200 nM for 24 h), a CCR5 antagonist. The antagonism of chemokines significantly reduced BMSC-induced upregulation of p65 (Figure 3), suggesting that upregulation p65 was related to BMSCs’ pain-relieving effect.

**Figure 4:**
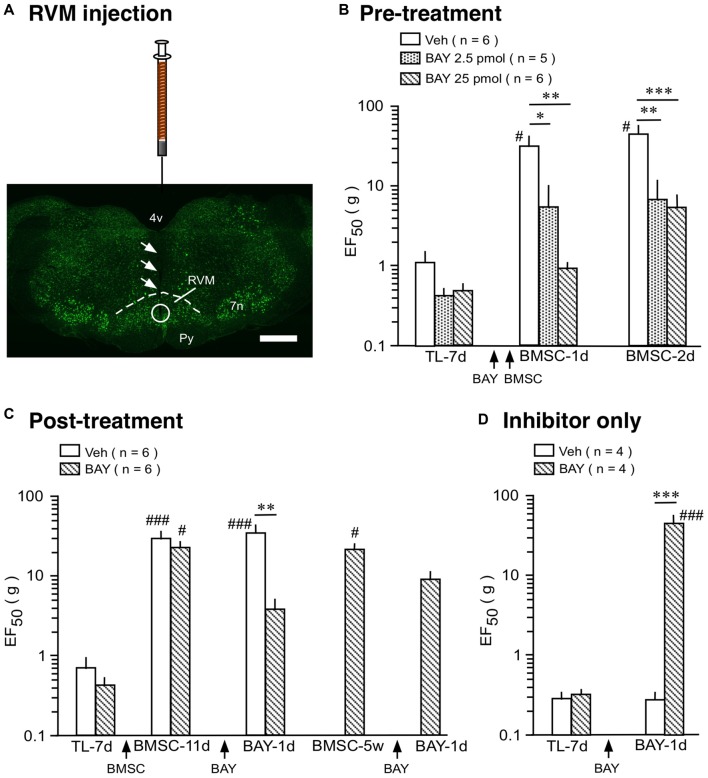
Effect of NF-κB activation inhibitor on BMSC-produced antihyperalgesia. **(A)** Image shows the injection site in RVM. Coronal brainstem sections were stained with green fluorescent Nissl stain. Arrows indicate the injection needle track and circle shows the site of injection. Scale = 1 mm. **(B)** Pre-treatment experiment, BAY 11-7082 (BAY) was injected at 2 h prior to BMSC infusion. **(C)** Post-treatment experiment, BAY was injected at 11 days and 5 weeks after BMSC infusion. **(D)** Inhibitor only experiment, BAY was injected at 7 days post-TL (TL-7d) and BMSCs were not infused. *p < 0.05, **p < 0.01, ***p < 0.001 vs. veh; ^#^*p*< 0.05, ^###^ *p* < 0.001 vs. TL-7d.

We then tested the effect of a selective NF-κB activation inhibitor, BAY 11-7082. In rats receiving TL, mechanical sensitivity was assessed as described (Ren, 1999; Guo et al., 2010). In the pre-treatment experiment, BAY 11-7082 (2.5 and 25 pmol/0.5 μl) or drug vehicle (0.5 μl) was injected at 2 h prior to BMSC infusion (Figures 4A,B). In vehicle-treated rats, EF_50_ was significantly increased following infusion of BMSCs, compared to 7 days post-TL (TL-7d) rats (Figure 4B; *p* < 0.05), indicating antihyperalgesia. Pretreatment with BAY 11-7082 significantly attenuated this antihyperalgesia (*p* < 0.05–0.001), examined at 1–2 days after BMSC infusion (Figure 4B). Post-treatment with BAY 11-7082 (25 pmol/0.5 μl) at 11 days after BMSC infusion led to a smaller reduction of EF_50_, compared to the pretreatment (Figures 4B,C). Injection of BAY 11-7082 at 5 weeks post-BMSC did not have an effect on BMSC-induced antihyperalgesia (Figure 4C).

Although NF-κB is seemingly involved in BMSC-induced protection as shown above, it is well known that activation of NF-κB pathway contributes to pro-inflammatory responses, characterized by induction of pro-inflammatory cytokines such as IL-1β (Ghosh et al., 1998; Bonizzi and Karin, 2004). Accordingly, NF-κB-involved signaling plays a role in enhanced pain sensitivity (Ross-Huot et al., 2013; Zhou et al., 2018). In our inhibitor only experiment, BAY 11-7082 (25 pmol/0.5 μl) was injected into the RVM at 7 days after TL and BMSCs were not infused. BAY 11-7082 significantly raised EF_50_s at 1 day (Figure 4D) and 2 days (not shown) after injection, indicating attenuation of TL-induced pain. This result is consistent with dual roles of NF-κB in pain hypersensitivity and BMSC-produced pain relief.

## Discussion

Our recent results indicate that interactions between infused BMSCs and the host immune system underlie their antihyperalgesic effect (Guo et al., 2017). Through the monocyte/macrophage population and associated chemokines and their receptors, BMSCs upregulate MOR in the RVM and produce potent antihyperalgesia. To extend these findings, we show here that the NF-κB pathway, a pivot of immune responses, is involved in BMSC’s pain-relieving effect. In the context of TL-induced orofacial hyperalgesia and transplantation of BMSCs, p65 of NF-κB was upregulated in the RVM and inhibition of NF-κB rekindled hyperalgesia.

The significance of upregulation of p65 in BMSCs’ antihyperalgesia is supported by its dependance on related chemokine signaling. Down regulation of CCL4 from BMSCs or blocking CCR2 and CCR5 of BMSCs prevented BMSCs from producing antihyperalgesia (Guo et al., 2017, 2018). Consistently, we show that p65 upregulation was significantly reduced under those conditions. However, the NF-κB pathway did not seem to be important for the late maintenance of pain-relieving effect of BMSCs. P65 was only upregulated at the first week after the BMSC treatment and pretreatment with NF-κB inhibitor was able to reverse antihyperalgesia. Contrastingly, post-treatment with BAY 11-7082 at the late 5-week time point was ineffective. These observations suggest that NF-κB activation is mainly involved in initialization of BMSCs’ therapeutic effect.

The present findings represent an apparent paradox with regard to function of NF-κB signaling. It is well known that NFκB activation is pro-inflammatory, characterized by induction of pro-inflammatory cytokines (Ghosh et al., 1998; Bonizzi and Karin, 2004). Studies have shown contribution of NF-κB to persistent pain (de Mos et al., 2009; Ross-Huot et al., 2013; Borghi et al., 2017). Thus, under persistent pain conditions without BMSC treatment, suppressing NF-κB should attenuate hyperalgesia (Ledeboer et al., 2005; Zhou et al., 2018). Without BMSCs, our results are consistent with pronociceptive role of NF-κB, as BAY 11-7082 attenuated mechanical hypersensitivity in TL rats.

It is appreciated that the function of NF-κB is diverse and dual roles of NF-κB signaling have been noticed. In fact, genes encoding proteins with opposite functions can be responsive to NF-κB (see Massa et al., 2006). NF-κB activity is also involved in the resolution of inflammation (Lawrence et al., 2001). Both pro- and anti-inflammatory cytokines can be induced by NF-kB activation (see Chang and Vancurova, 2013). We have observed upregulation of IL-10 and CD206, a marker of anti-inflammatory microglia, in the RVM after BMSC injection in TL rats (Ren, in press), which correlates with the increase of p65. NF-κB has been shown to positively regulate Foxp3 expression in Tregs (T regulatory cells; Long et al., 2009), which is in line with its role in the resolution of inflammation. RelB of NF-κB can induce and suppress gene expression (Madge and May, 2011). In the multiple sclerosis model, the activation of NF-κB protects oligodendroctyes against inflammation (Stone et al., 2017). The antihyperalgesic effects of NF-κB could be explained by phenotype switching action of BMSCs on immune cells. BMSCs can reprogram monocyte/macrophage and promote an anti-inflammatory phenotype (Al-Hanbali et al., 2009; Németh et al., 2009; Dayan et al., 2011; Guo et al., 2017), likely involving NF-κB activation (Pilette et al., 2010). Together with endogenous opioids, BMSC-produced antihyperalgesia should offset the pain facilitatory effect of NF-κB.

We noted that p65 of NF-κB widely distributed in RVM neurons, which provides an integrated platform for interactions between the neuron and immune system. We have shown that RVM MOR-containing neurons contribute to BMSC-produced antihyperalgesia (Guo et al., 2011, 2017). MOR activates NF-κB signaling (Hou et al., 1996; Wang et al., 2004; Liu and Wong, 2005). Upregulation of MOR expression in neurons may also be downstream to NF-κB activation (Kraus et al., 2003; Wei and Loh, 2011; Wagley et al., 2013). In cell cultures, TNF stimulates MOR expression in immune cells as well as neurons involving the NF-κB pathway (Kraus et al., 2003). Additionally, through the NF-κB pathway, TNF promoted IL-1receptor antagonist release from mesenchymal stromal cells to facilitate wound healing (Kou et al., 2018). Interestingly, compared to primary BMSCs, TNF is dramatically down-regulated in 20P BMSCs that are ineffective in producing antihyperalgesia (Guo et al., 2017) and TNF activate NF-κB pathway through phosphorylation of p65 in HeLa cell cultures (Wang and Baldwin, 1998). On the other hand, IL-6 induces MOR transcription in the human neuroblastoma cell line that involves transcription factors signal transducers and activators of transcription 1 (STAT1) and STAT3, but not NF-κB (Börner et al., 2004). It would be interesting to know cytokine-specific NF-κB-mediated regulation of MOR *in vivo* after transplantation of BMSCs.

**Figure 5:**
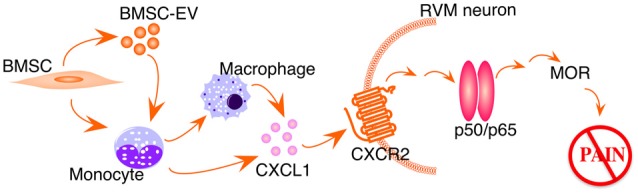
Schematic illustration of BMSC-induced activation of NF-kB pathway. EV, extracellular vesicles. See text for details.

Studies have shown that CXCL1-CXCR2 chemokine signaling produces biological effects involving downstream NF-κ activation (Cai et al., 2010; Dong et al., 2013). We speculate that CXCL1-CXCR2 signaling induces NF-κB upregulation in our model (Figure 5). Systemic BMSCs interact directly, or via their secreted extracellular vesicles, with monocytes in the circulation. Removing monocytes/macrophages largely attenuates BMSCs’ antihyperalgesia (Guo et al., 2017) and monocytes have been shown as major recipient of BMSC-derived extracellular vesicles (Di Trapani et al., 2016). In TL rats receiving BMSC treatment, CXCL1 is specifically upregulated in peripheral monocytes and cerebrospinal fluid, and CXCR2 is upregulated in RVM neurons containing MOR (Guo et al., 2017). Further, the CXCL1-CXCR2 signaling in RVM neurons is required for BMSC-produced antihyperalgesia (Guo et al., 2017). It remains to be shown that BMSC-induced upregulation of MOR is causally related to NF-κB activation involving the CXCL1-CXCR2 axis.

The present results extend our previous findings that *in vivo* immune interactions underlie mechanisms of BMSC-produced pain relief. We provide first evidence that the NF-κB signaling pathway in the descending circuitry is involved in BMSC-produced behavioral antihyperalgesia. The activation of NF-κB following BMSCs leads to a protective outcome that opposes to a conventional proinflammatory role. The results suggest that activation of NF-κB may have diverse functional consequences, depending upon the type of stimuli and cellular environment. Future studies should explore cell-specific involvement of different NF-κB dimers, canonical and non-canonical (Massa et al., 2006), under different pathological and therapeutic conditions.

## Author Contributions

WG: conception and design, collection and assembly of behavioral and western blot data, data analysis and interpretation, manuscript writing. SI: conception and design, BMSC cultures, data analysis and interpretation, manuscript writing. J-LY: collection and assembly of immunohistochemistry data. SZ: collection and assembly of behavioral data. HL: collection and assembly of western blot data. HX: BMSC procedure and design. KM: design and data analysis. RD: conception and design, data analysis and interpretation, manuscript writing. FW: conception and design, data analysis and interpretation, manuscript writing. KR: conception and design, assembly of data, data analysis and interpretation, manuscript writing. All authors gave final approval of the manuscript.

## Conflict of Interest Statement

The authors declare that the research was conducted in the absence of any commercial or financial relationships that could be construed as a potential conflict of interest.
